# *Yersinia enterocolitica* in Italy: A Case of Septicemia and Abdominal Aortic Aneurysm Infection

**DOI:** 10.3389/fmed.2018.00156

**Published:** 2018-05-24

**Authors:** Donatella M. Rodio, Alessia Bressan, Cecilia Ambrosi, Daniela Scribano, Rita Tolli, Wassim Mansour, Francesco Speziale, Guido Antonelli, Maria Trancassini, Valeria Pietropaolo

**Affiliations:** ^1^Department of Public Health and Infectious Diseases, Sapienza University of Rome, Rome, Italy; ^2^Dani Di Giò Foundation–Onlus, Rome, Italy; ^3^Istituto Zooprofilattico Sperimentale del Lazio e della Toscana Mariano Aleandri, Rome, Italy; ^4^Vascular and Endovascular Surgery Division, Department of Surgery Paride Stefanini, Policlinico Umberto I, Sapienza University of Rome, Rome, Italy; ^5^Laboratory Affiliated to Istituto Pasteur Italia-Fondazione Cenci Bolognetti, Department of Molecular Medicine, Sapienza University of Rome, Rome, Italy

**Keywords:** *Yersinia enterocolitica*, septicemia, aortic aneurysm, serogroup O:9, virulence factors

## Abstract

We report a case of *Yersinia enterocolitica* septicemia in a 63-year-old patient admitted to the Vascular Surgery Department of Umberto I Hospital (Rome, Italy) for an abdominal aortic aneurysm. The microorganism, recovered from both peripheral blood cultures and aneurysmatic aortic wall specimens, was identified as *Y. enterocolitica* using matrix-assisted laser desorption ionization–time of flight analysis (MALDI-TOF MS) and 16S rDNA gene sequencing. The isolate responsible for septicemia belonged to the O:9 serotype (biogroup 2). A genetic screening of the isolate made it possible to detect the presence of both the *yst* and *ail* genes, encoding a heat-stable enterotoxin and a protein involved in invasion/adherence and serum resistance, respectively. Our case contributes in enriching epidemiological data concerning *Y. enterocolitica* infections, which might represent severe complications in patients suffering from cardiovascular diseases. Moreover, this study, together with the others, should be regarded as valuable and useful tools for monitoring the rate of infections worldwide.

## Introduction

*Yersinia enterocolitica* is a non-spore Gram-negative bacterium belonging to *Yersinia* genus and to the *Enterobacteriaceae* family. Among *Yersinia* species, only *Y. pestis, Y. pseudotuberculosis*, and some *Y. enterocolitica* strains are pathogenic for humans, whereas others are environmental species that might be opportunistic pathogens. *Y. enterocolitica* is a heterogeneous group of bacterial strains, classified into 6 biogroups (1A, 1B, 2, 3, 4, and 5) based on phenotypic characteristics, human/animal pathogenicity, and ecologic and geographic distribution (1). Serologically, *Y. enterocolitica* is further distinguished into more than 57 O serogroups, depending on their lipopolysaccharide-O-antigens. Only isolates belonging to biogroup 1A may be considered avirulent so far. Among the other 5 biogroups, strains belonging to serogroups O:3 (biogroup 4), O:5,27 (biogroups 2 and 3), O:8 (biogroup 1B), and O:9 (biogroup 2) are most commonly isolated from human samples worldwide (2, 3). However, the most prevalent *Y. enterocolitica* serogroup in many European countries is serogroup O:3 followed by O:9, whereas the serogroup O:8 is mainly detected in the United States (1, 3). Despite a significant decrease between 2008 and 2016, human Yersiniosis represents the third most commonly reported bacterial food-borne zoonosis in all EU countries (https://ecdc.europa.eu/).

As a foodborne pathogen, *Y. enterocolitica* causes acute terminal ileitis and mesenteric Iymphadenitis. Depending on both patient conditions and bacterial serogroup, it can spread at the systemic level (3, 4). Several septic complications have been described that can evolve, eventually, into endocarditis or infected aortic aneurysm, also known as mycotic aneurysm (3). At present, only 14 papers associated with *Y. enterocolitica* have been reported in literature (Table 1), including 13 cases of infected abdominal aortic aneurysms (AAA). The main pathogenic properties of *Y. enterocolitica* are due to the presence of the two chromosomally encoded genes, *ail* and *yst* (1, 19, 20). The *ail* gene encodes for the membrane-associated protein Ail, involved in adhesion to and invasion into eukaryotic cells, as well as serum resistance. Conversely, the *yst* gene encodes for thermostable enterotoxin Yst, whose presence is related with diarrheal illness, although its role remains largely controversial (21). Being the most common virulence genes of *Y. enterocolitica*, both are considered excellent PCR targets for bacterial detection and subtyping as well as for its pathogenicity potential (22).

**Table 1:** Data summarizing previously reported cases of *Y. enterocolitica* mycotic aneurysms.

**References**	**Age**	**Sex**	**First isolated from**	**GI symptoms**	**Endocarditis**	**Site of aneurysm**	**Serogroup/Biogroup**	**Antibiotic therapy**	**Outcome**
(5)	53	M	Stool and blood	Yes	No	Carotid artery	Biotype 4	Cefamandole	Good
(6)	79	M	Blood	Yes	No	AAA^*^	Serotype O:3, Biotype 4	Ampicillin and gentamicin	Fatal
(7)	76	M	Aneurysm wall	Yes	No	AAA^*^	Serotype O:9, Biotype 2	Gentamicin and Co-trimoxazole	Good
8	59	M	Blood	Yes	No	AAA^*^	Biotype 4	Cefuroxime and metronidazole	Fatal
(9) (6 cases)	73	M	Blood	No	No	AAA^*^ and Popliteal Artery	Serotype O:9	Ciprofloxcin and Co-trimoxazole	Fatal (2/6)
(10)	57	M	Resected aorta	No	No	AAA^*^	Serotype O:3, Biotype 4	Ciprofloxacin and aztreonam	Fatal
(11) (3 cases)	70	M	Blood	No	No	AAA^*^	Serotype O:9	Ciprofloxcin /tetracycline and Ceftriaxone	Fatal (2/3)
(12)	64	M	Blood	Yes	No	Femoral artery and AAA^*^	Unspecified	Unspecified	Fatal
(13)	78	M	Blood	No	No	AAA^*^	Serotype O:9, Biotype 2	Ofloxacin	Good
(14)	55	M	Blood	No	No	AAA^*^	Serotype O:3, Biotype 4	Gentamicin and ceftriaxone	Fatal
15	70	M	Vascular graft	No	No	AAA^*^	Serotype O:3, Biotype 4	Cefotaxime and ciprofloxacin	Good
(16)	74	M	Blood	Yes	Yes	AAA^*^	No serogroup/Biotype 2	Amoxicillin, metronidazole and gentamicin	Good
(17)	78	M	Blood	No	Yes	AAA^*^	Unspecified	Ciprofloxacin	Good
(18)	68	M	Blood	No	No	AAA^*^	Unspecified	Piperacillin-tazobactam	Good

* *AAA, abdominal aortic aneurysms*.

Herein, we describe a case of *Y. enterocolitica* septicemia in a patient suffering from an AAA Microbiological and molecular analyses have demonstrated the presence of a strain belonging to the O:9 serotype, carrying both the *ail* and *yst* genes and resistant to amoxicillin/clavulanate.

## Case report

### Ethics statement

The subject gave a written informed consent in accordance with the Declaration of Helsinki.

A 63-year-old patient was admitted to the Vascular Surgery Department of Umberto I Hospital (Rome, Italy) for an AAA.

**Figure 1:**
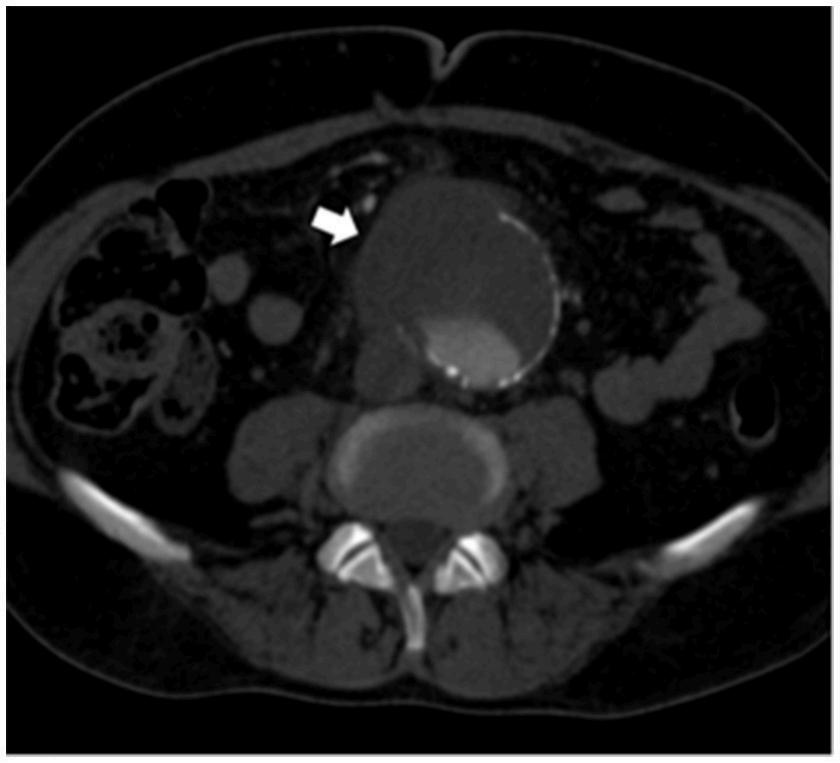
Preoperative CT scan showing an abdominal aortic aneurysm surrounded by inflammation/fluid collection (white arrow).

**Figure 2:**
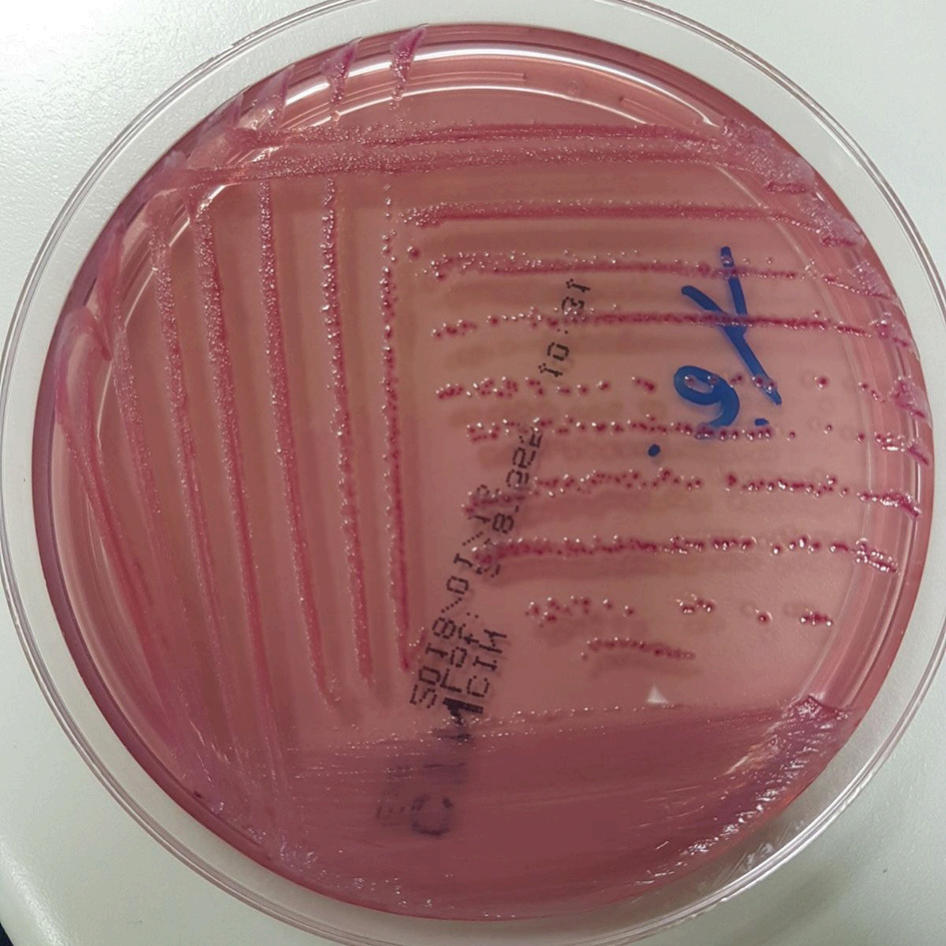
*Y. enterocolitica* on CIN agar: small colonies with the typical red bull's eye appearance, namely a red center with colorless translucent rim.

**Figure 3:**
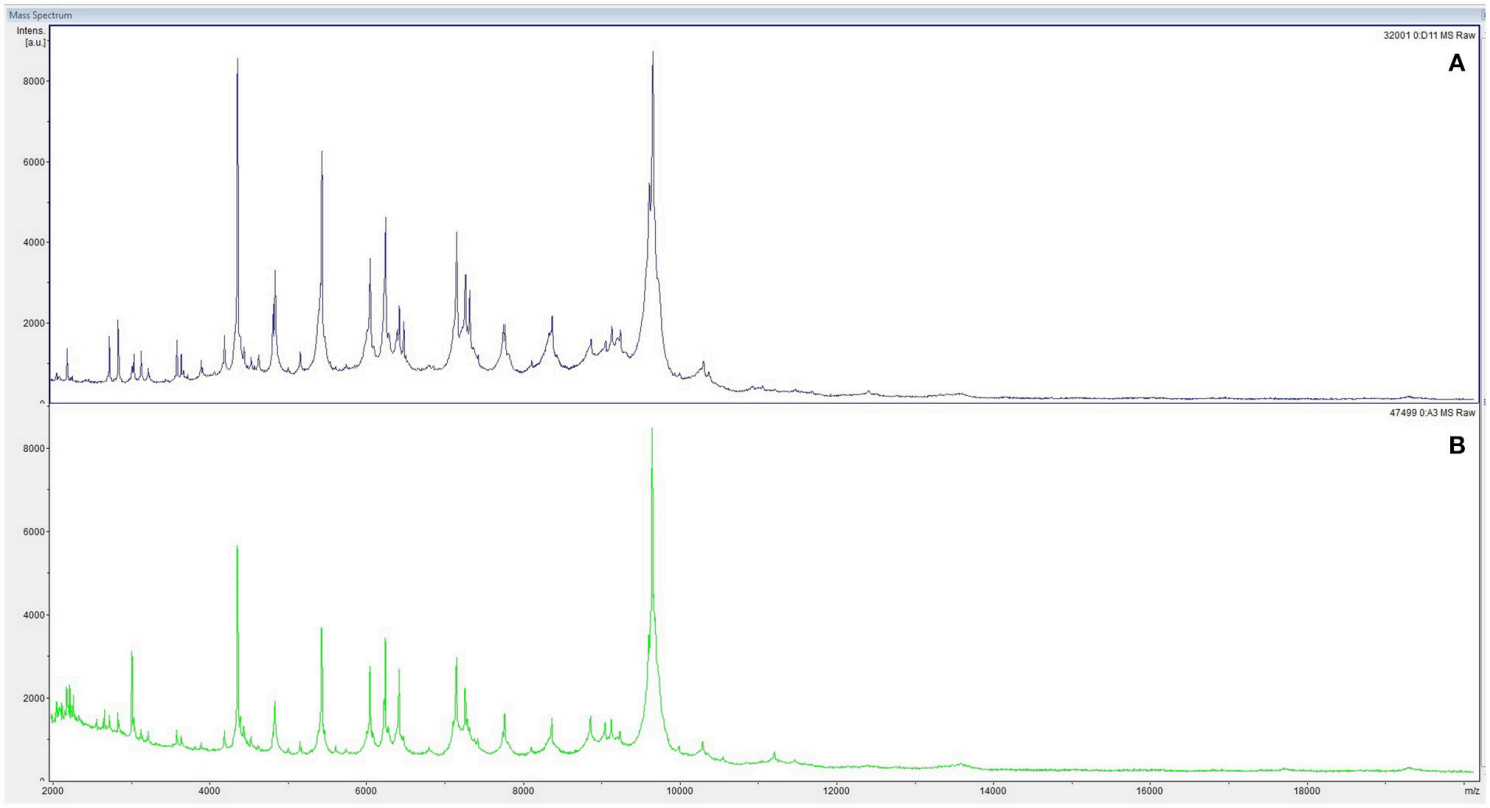
Peptide mass fingerprinting of *Y. enterocolitica* from peripheral blood cultures **(A)** and from aneurysm specimens **(B)** by MALDI-TOF MS.

At admission, the patient presented with low-grade fever (37.2°C) and reported a history of asthenia and weight loss in the previous months. No abdominal symptoms, vomiting or diarrhea were referred. In September 2017, the patient performed an abdominal ultrasound and blood tests, including three sets of peripheral blood culture that were sent to the Microbiology laboratory. Blood tests showed an erythrocyte sedimentation rate of 48 mm/h, a white blood cell (WBC) count of 13.94 × 10^9^/L and a C-reactive protein (CRP) level of 185 mg/L. An empirical therapy was therefore started with the intravenous administration of daptomycin (500 mg die), ertapenem (1 g die), and fluconazole (400 mg die). Abdominal ultrasound identified described an abdominal aortic aneurysm with a diameter of 6.42 cm. The computed tomography (CT) scan confirmed the presence of the AAA with a periaortic inflammation (Figure 1). Moreover, transthoracic echocardiogram ruled out active endocarditis. The patient therefore underwent endovascular repair. After 24 h of incubation in the automatic Virtuo BacT/ALERT (bioMérieux, Inc. France), two sets of blood cultures became positive. Microscopic examination revealed motile Gram-negative coccobacilli. Colonies on MacConkey agar were small, round and non-lactose fermenting. Colonies were subcultured onto CIN agar plates (Cefsulodin Irgasan Novobiocin) (bioMérieux, Inc. France), selective for *Yersinia* spp. After 24 h of incubation at 28°C, the colonies displayed the typical red bull's eye appearance, characterized by a red center with a colorless translucent rim (Figure 2). Biochemical tests of the colonies, using VITEK 2 System (bioMérieux, Inc. France), identified *Y. enterocolitica/frederiksenii* with 99% probability. Accordingly, MALDI-TOF MS System confirmed the identification of *Y. enterocolitica*, with a score of 2.265 (23) (Figure 3). Finally, the 16S rDNA gene sequence analysis confirmed the *Y. enterocolitica* identification. Antibiotic susceptibility tests with VITEK 2 System (bioMérieux, Inc. France) and MICROSCAN WalkAway System 96 Plus (Beckman Coulter S.r.l.) revealed that the *Y. enterocolitica* isolate was susceptible to all antibiotics tested, except to amoxicillin/clavulanate (Table 2). Serogrouping analysis using specific *Y. enterocolitica* antisera evidenced that the *Y. enterocolitica* isolate belonged to the O:9 serotype (biogroup 2). Finally, the presence of both *ail* and *yst* genes in the *Y. enterocolitica* isolate was detected using Real-time PCR.

**Table 2:** Antibiotic susceptibility MICs (expressed as mg/L) of the *Y. enterocolitica* isolate.

**Antibiotic**	**MIC VITEK2**	**MIC MICROSCAN**	**EUCAST 2017**
Amikacin	4	≤8	S
Amoxicillin/Clavulanate	16	16	R
Cefepime	≤1	≤1	S
Cefotaxime	≤1	≤1	S
Ceftazidime	≤1	≤1	S
Colistin	≤0.5^*^	≤2	S
Ciprofloxacin	≤0.25	≤0.5	S
Ertapenem	≤0.5	≤0.5	S
Fosfomycin	≤16	≤32	S
Gentamicin	≤1	≤2	S
Imipenem	≤0.25	≤1	S
Meropenem	≤0.25	≤1	S
Tigecycline	≤0.5	≤1	S

* *The result of Colistin obtained with the VITEK2 method has a high percentage of Very Major Error as reported by the company*.

Two weeks after the endovascular procedure, the patient underwent open surgical repair. During the procedure, the surgeon reported a strong foul necrotic smell and noticed tight adhesions between the inflamed small intestine and the aneurysm. Specimens were collected from the aneurysmatic aortic wall and sent to the Microbiology laboratory. These aneurysm specimens were, firstly, incubated in brain heart infusion (BHI) broth and, subsequently, plated on different microbiological media, including CIN agar plates. Again, microbiological, biochemical, molecular, antibiotic susceptibility, and serogrouping analyses identified the clinical isolate as *Y. enterocolitica* O:9 serotype (biogroup 2). Therefore, we concluded that the same *Y. enterocolitica* isolate was colonizing the patient. The antibiotic therapy was modified; the patient suspended daptomycin and fluconazole and was treated intravenously with ertapenem (1 g die) and tigecycline (100 mg for first day and 50 mg for following days) for 4 weeks. Over this period, all the sets of blood culture were negative. The patient was discharged home 8 weeks after surgery.

## Materials and methods

Peripheral blood culture bottles, for both aerobic and anaerobic microorganisms, were incubated for 24 h in the automatic Virtuo BacT/ALERT (bioMérieux, Inc. France). Aneurysm specimens were incubated in BHI at 37°C for 24 h. Clinical isolates were examined under light microscope (400x) for motility and upon Gram staining. In parallel, isolates were seeded on Blood agar, MacConkey agar, Extended Spectrum Beta-Lactamase (ESBL) agar (bioMérieux, Inc. France), and CIN agar plates and incubated at 28°C for 24 h. Colonies were identified by VITEK 2 System (bioMérieux, Inc. France), by MALDI-TOF MS System (Bruker Daltonics, Inc.) and by 16S rDNA gene sequencing, as previously described respectively in Febbraro et al. (24) and in Yu et al. (25). Antimicrobial susceptibility testing was performed by VITEK 2 System (bioMérieux, Inc. France) and by MICROSCAN WalkAway System 96 Plus (Beckman Coulter S.r.l.). The resulting minimum inhibitory concentration (MIC) values were classified into clinical categories of susceptible, intermediate or resistant, following the EUCAST recommendations (26). Serogrouping analysis was performed by slide agglutination using O:3, O:5, O:8, and O:9 antisera (Biogenetics, s.r.l. Italy) following the recommendation of the manufacturer. Real-time PCR was performed as previously described (21). Both analyses were carried out at the Experimental Zooprophylactic Institute of Lazio and Tuscany “Mariano Aleandri” (Rome, Italy).

## Discussion

There has been an increasing body of evidence showing that *Y. enterocolitica* serogroups O:3 and O:9 are a cause of mycotic aneurysm and septicaemia, mainly in males over 50 years, with risk factors for cardiovascular disease (10, 14). Herein, we report a case of *Y. enterocolitica* serogroup O:9 (biogroup 2) septicemia in a patient with an AAA. It is important to note that this is the first study reporting the identification of *Y. enterocolitica* both in the peripheral blood and in the specimens collected from the aneurysmatic aortic wall.

It has been reported that, following ingestion, virulent *Y. enterocolitica* crosses the intestinal mucus layer and binds to the M cells overlying Peyer's Patches. Thereafter, bacteria penetrate these cells to gain access to and multiply in subjacent tissue. Among others, attachment and invasion of M cells is promoted by the membrane-associated Ail protein, encoded by the chromosomal invasion *ail* gene, thereby facilitating the spread to several tissues, as well as bloodstream (1). Also in this environment, Ail provides strong protection to complement killing bactericidal activity, thereby allowing survival of bacteria in the blood and increasing its spreading (1). Due to *Y. enterocolitica* predilection for vascular tissues, it is highly likable that bacteria colonize the aneurysmatic aorta during bacteremia. On the other hand, the thermostable enterotoxin Yst, encoded by the *yst* gene, is mainly related with diarrheal illness (21). Although its role remains largely controversial, it has been reported in an animal model that Yst can cause weight loss without the occurrence of diarrhea, possibly depending on the degree of host immune competency.

Our clinical and microbiological data support the hypothesis that *Y. enterocolitica* firstly colonized the patient's intestine. Accordingly, the patient referred a weight loss in the previous months. Thereafter, the inflammatory process of the small intestine, in tightly conjunction to the aneurysm, allowed *Y. enterocolitica* translocation into the aneurysm, thereby shortening the period of bacteraemia. *Y. enterocolitica* septicemia occurs commonly in patients suffering from underlying disease(s) (1). Although our patient did not seem to have predisposing factors, the high degree of inflammation of the small intestine might have even favored *Y. enterocolitica* translocation into the aneurysm.

## Concluding remarks

Our case contributes in enriching epidemiological data concerning *Y. enterocolitica* infections, which might represent severe complications in patients suffering from cardiovascular diseases. The most common site of infections in these patients is the aortic aneurysm, causing the mycotic aneurysm. Therefore, colonization of the aneurysmatic aorta during bacteraemia can lead to vessel rupture due to inflammation and necrosis, but also to endocarditis, due to the contiguity of the infection site (1, 18). Finally, together with the previous reports, our case underlines the importance of the microbiological surveillance of *Y. enterocolitica*. Regretfully, no routine screening test for detecting *Y. enterocolitica* for cardiovascular disease patients is available at the moment. No reliable data of monitoring circulating *Yersinia* spp. on food and animals are available, since it is not mandatory for Zooprophylactic Institutes to report data (27). Therefore, all case reports on *Y. enterocolitica* involved in cardiovascular diseases should be regarded as valuable and useful tools to monitor the rate of infections worldwide.

## Author contributions

DR, AB, CA, MT, and VP contributed equally to this work. DR, AB, MT, and VP collected clinical data and guided the testing and experiments. DS and CA guided the 16S rDNA gene analyses for bacterial identification. RT conducted the serogroup analysis using specific antisera and Real-time PCR to detect virulence genes. WM and FS collected clinical samples and provided clinical data. All of authors discussed the results and implications. DR, AB, MT, DS, CA, and VP wrote the manuscript. All the authors revised and approved the final manuscript.

### Conflict of interest statement

The authors declare that the research was conducted in the absence of any commercial or financial relationships that could be construed as a potential conflict of interest. The reviewer MF declared a shared affiliation, though no other collaboration, with several of the authors DR, AB, CA, DS, MT, and VP to the handling Editor.
